# Hospital antimicrobial stewardship: profiling the oral microbiome after exposure to COVID-19 and antibiotics

**DOI:** 10.3389/fmicb.2024.1346762

**Published:** 2024-02-27

**Authors:** Patricia Buendia, Krystal Fernandez, Castle Raley, Ali Rahnavard, Keith A. Crandall, Jose Guillermo Castro

**Affiliations:** ^1^Lifetime Omics, Miami, FL, United States; ^2^The George Washington University Genomics Core, Milken Institute School of Public Health, The George Washington University, Washington, DC, United States; ^3^Department of Biostatistics and Bioinformatics, Computational Biology Institute, Milken Institute School of Public Health, The George Washington University, Washington, DC, United States; ^4^Division of Infectious Diseases, Leonard M. Miller School of Medicine, University of Miami, Miami, FL, United States

**Keywords:** broad-spectrum antibiotics, COVID-19, sepsis, saliva microbiome, *Candida albicans* (*C. albicans*), *Staphylococcus aureus* (*S. aureus*), hospital antimicrobial stewardship

## Abstract

**Introduction:**

During the COVID-19 Delta variant surge, the CLAIRE cross-sectional study sampled saliva from 120 hospitalized patients, 116 of whom had a positive COVID-19 PCR test. Patients received antibiotics upon admission due to possible secondary bacterial infections, with patients at risk of sepsis receiving broad-spectrum antibiotics (BSA).

**Methods:**

The saliva samples were analyzed with shotgun DNA metagenomics and respiratory RNA virome sequencing. Medical records for the period of hospitalization were obtained for all patients. Once hospitalization outcomes were known, patients were classified based on their COVID-19 disease severity and the antibiotics they received.

**Results:**

Our study reveals that BSA regimens differentially impacted the human salivary microbiome and disease progression. 12 patients died and all of them received BSA. Significant associations were found between the composition of the COVID-19 saliva microbiome and BSA use, between SARS-CoV-2 genome coverage and severity of disease. We also found significant associations between the non-bacterial microbiome and severity of disease, with *Candida albicans* detected most frequently in critical patients. For patients who did not receive BSA before saliva sampling, our study suggests *Staphylococcus aureus* as a potential risk factor for sepsis.

**Discussion:**

Our results indicate that the course of the infection may be explained by both monitoring antibiotic treatment and profiling a patient’s salivary microbiome, establishing a compelling link between microbiome and the specific antibiotic type and timing of treatment. This approach can aid with emergency room triage and inpatient management but also requires a better understanding of and access to narrow-spectrum agents that target pathogenic bacteria.

## Introduction

The oral cavity is one of the entry points in the body for SARS-CoV-2. SARS-CoV-2 is known to bind to ACE2 receptors which are highly expressed in the human oral tissue that comprise the gingiva, tongue, and palate (Okui et al., 2021). Studies have shown that dysbiosis of the local airway microbiome induced by SARS-CoV-2 initially occurs in the oral cavity and subsequently impacts distant microbiomes across connected body sites via the oral-lung or oral-gut axis (Bao et al., 2020; Xu et al., 2020; Hazan et al., 2022). A 2021 study found that oropharyngeal microbiota alterations were associated with COVID-19 severity (Ma et al., 2021). As human saliva contains a large amount of human DNA and RNA compared to stool and other specimens (Armstrong et al., 2021), analysis of its microorganisms has received less attention. A recent COVID-19 saliva microbiome study focused on methods to deplete human DNA while preserving the microbiome composition of samples (Armstrong et al., 2021). Moreover, during the COVID-19 pandemic, a preference was to use nasopharyngeal swabs for PCR testing to detect SARS-CoV-2, and most COVID-19 oral microbiome studies have used swabs instead of saliva as a consequence.

A common practice during the COVID-19 outbreak was to treat patients admitted to the hospital for a possible concomitant bacterial pneumonia, at least for a few days until additional test results excluded bacterial pneumonia. In addition, broad-spectrum antibiotics (BSA) were prescribed for patients diagnosed with or at risk of sepsis or septic shock. While many COVID-19 studies have focused on epidemiological associations of SARS-CoV-2 (Rahnavard et al., 2021) and dynamics of microbiome during infection, CLAIRE (CLuster AI pREdiction of disease progression) is the only study to investigate the COVID-19 saliva microbiome in hospitalized patients with a focus on sepsis and BSA (see section “Medical records and outcome analysis”). The SARS-CoV-2 virus was characterized through Illumina’s Respiratory Viral Oligo Panel (RVOP) sequencing (Illumina). Our study includes a comparison with 5 healthy subjects from a saliva metagenomic study (Armstrong et al., 2021). We selected saliva as a biospecimen as it is easy to collect at any time compared to stool samples and has not been studied enough in the context of infectious disease and sepsis. We were able to validate our hypothesis that the saliva microbiome can be used as a biomarker for infection disease severity with suggested guidelines on how to incorporate the effect and timing of broad-spectrum antibiotics into a prediction model.

## Materials and methods

### Patient enrollment

The inclusion criteria for our study were patients at age 18 years and older and admitted to the University of Miami (UM) hospital with a clinically diagnosed COVID-19. COVID-19 infection was determined using one of 3 diagnostics (SARS-CoV-2 NAAT, COVID-19 RT PCR, COVID-19 ACCULA NAAT), all of which can be classified to be at least 95% accurate. Upon obtaining informed consent, participants were asked to provide a one-time saliva sample. The study was approved by the University of Miami IRB ID: 20200724.

### Study design and sample collection

Saliva samples were collected after patient enrollment. Instructions to patients were not to eat, drink, smoke, or chew gum for 15 min before giving a sample. The Zymo R1210-E–DNA/RNA Shield Saliva Collection Kit–DX, a 510(k)-Cleared FDA Class II Medical Device, was used to collect the saliva in virus inactivating and preserving DNA/RNA Shield buffer, allowing storage at room temperature for up to 3 weeks. Samples were batched and stored at room temperature and shipped in ice every 2–2.5 weeks to the George Washington University Genomics Core for storage at −20 degrees prior to sequencing. Supplementary Figure 1 illustrates the study workflow.

### Medical records and outcome analysis

Medical records were collected for all patients (Table 1). Per hospital COVID-19 management protocol, all patients were started on ceftriaxone and azithromycin when they were admitted to the hospital with clinical suspicion of COVID-19. Patients may have received additional antibiotics if the hospital team deemed it necessary (e.g., doxycycline hyclate used to treat 6 patients). Also, per hospital protocol, patients who met the criteria for sepsis were treated with broad-spectrum sepsis antibiotics (BSAs), specifically vancomycin and one of the following: cefepime, meropenem, or zosyn (piperacillin/tazobactam) (Table 2).

**TABLE 1:** Patients’ characteristics.

	Critical (*n* = 25)	Moderate (*n* = 95)		Critical (*n* = 25)	Moderate (*n* = 95)
**Age**			**BMI**		
Median (range)	60 (34–91)	54 (19–86)	< 18.5 (underweight)	0 (0%)	3 (3.16%)
0–19 (%)	0 (0%)	1 (1.05%)	18.5–24.9 (normal weight)	1 (4.00%)	14 (14.74%)
20–29 (%)	0 (0%)	5 (5.26%)	25–29.9 (overweight)	5 (20.00%)	22 (23.16%)
30–39 (%)	3 (12.00%)	17 (17.89%)	30–34.9 (obesity Class I)	7 (28.00%)	25 (26.32%)
40–49 (%)	3 (12.00%)	15 (15.79%)	35–39.9 (obesity Class II)	7 (28.00%)	19 (20.00%)
50–59 (%)	6 (24.00%)	23 (24.21%)	> 40 (obesity Class III)	4 (16.00%)	11 (11.58%)
60–69 (%)	7 (28.00%)	16 (16.84%)	Unknown	1 (4.00%)	1 (1.05%)
70–79 (%)	2 (8.00%)	14 (14.74%)	**Comorbidities**		
80–100 (%)	4 (16.00%)	4 (4.21%)	Diabetic	12 (48.00%)	28 (29.47%)
**Sex**			CKD	6 (24.00%)	13 (13.68%)
Female	13 (52.00%)	44 (46.32%)	COPD	5 (20.00%)	14 (14.74%)
Male	12 (48.00%)	51 (53.68%)	Cancer	5 (20.00%)	23 (24.21%)
**Ethnicity**			HD	14 (56.00%)	29 (30.53%)
Hispanic or Latino	15 (60.00%)	49 (51.58%)	Obesity	7 (28.00%)	29 (30.53%)
African American	1 (4.00%)	1 (1.05%)	**Blood result median (range)**		
White	13 (52.00%)	45 (47.37%)	Leukocyte count (×10^∧^3/μl, 4.00–10.50)	7.83 (3.04–18.63)	6.92 (0.45–15.25)
Other or unknown	1 (4.00%)	3 (3.16%)	Platelets (×10^∧^3/μl, 140–400)	225.96 (113.5–397.5)	231.97 (11–611.75)
Non-Hispanic or Latino	9 (36.00%)	44 (46.32%)	Hemoglobin (g/dL, male = 13.3–16.3, female = 11.1–14.6)	12.59 (8.325–15.55)	11.87 (6.77–15.5)
African American	8 (32.00%)	28 (29.47%)	C-reactive protein (mg/dL, 0–0.5)*	9.88 (0.93–29.5)	6.88 (0.3–31.2)
White	1 (4.00%)	15 (15.79%)	Alanine aminotransferase (U/L, male = 0–41, female = 0–33)	42.02 (13.33–115.33)	53.29 (7.5–323.33)
Other or unknown	0 (0%)	1 (1.05%)	Lactose dehydrogenase (U/L, male = 135–225, female = 135–214)*	456.44 (199.67–720)	385.85 (142–1,578.5)
Ethnicity unknown	1 (4.00%)	2 (2.11%)	D-dimer (ug/ml, < 0.50)	2.06 (0.4–10.8)	2.4 (0.3–18.45)
African American	0 (0%)	2 (2.11%)	NLR (ratio of neutrophils to lymphocyte)*	7.37 (1.04–19.9)	6.65 (0.63–81.74)
Other or unknown	1 (4.00%)	0 (0%)	LMR (ratio of lymphocyte to monocyte)	2.46 (0.75–7.5)	3.1 (0.54–34.5)
			WBC count (×10^∧^3/μl, 4–10.50)	7.83 (18.63–2.04)	6.93 (15.25–0.45)

*Indicates a significant difference between the 2 severity groups.

**TABLE 2:** Broad-spectrum sepsis antibiotics (BSA) treatment categorization.

	Critical (*n* = 25)	Moderate (*n* = 95)	Survived (*n* = 108)	Expired (*n* = 12)
Early broad-spectrum sepsis antibiotics (early BSA)*				
Before sampling	2 (8.00%)	18 (18.95%)	20 (18.52%)	0 (0%)
Same-day of sampling	1 (4.00%)	7 (7.37%)	8 (7.4%)	1 (8.33%)
No early broad-spectrum sepsis antibiotics (no early BSA)*				
After sampling	19 (76.00%)	6 (6.32%)	14 (12.96%)	11 (91.67%)
Never prescribed	3 (12.00%)	64 (67.37%)	67 (62.04%)	0 (0%)

*BSA treatment categorization with two main groups based on the timing of sepsis diagnosis before or after saliva sampling. The “Early BSA” group was split into before and same-day saliva sampling. The “No early BSA” group was split into BSA’s “After Sampling” and “Never Prescribed”. BSA treatment: vancomycin (glycopeptide), cefepime (cephalosporin), meropenem (carbapenem), zosyn (beta-lactamase inhibitor).

Medical records identified 4 patients with a false positive diagnosis of COVID-19 as they had negative COVID-19 PCR tests. One was reclassified and another was added as a false positive. SARS-CoV-2 molecular test results were retrieved from the medical records. For this study, we used the first measurement taken upon admission.

Sepsis was diagnosed with the following elements: 3 main criteria with other sub-criteria. Sepsis is diagnosed if there is (1) clinical suspicion of infection, and (2) either (a) (SBP < 90, MAP < 65, or decrease in SBP > 40 from baseline), or (b) evidence of > 1 organ dysfunction, Creatinine > 2, TBili > 2, PLT < 100, Lactate > 2, IR > 1.5, PTT > 60, and (3) at least 2 of these are present: (a) Temp < 96.8°F (36°C) or > 100.9°F (39.30°C), (b) HR > 90, (c) RR > 20 or PaCO2 < 32, (d) WBC > 12K, or < 4K, or > 10% Bands.

The severity of the COVID-19 disease was categorized using the Adaptive COVID-19 Treatment Trial (ACTT) COVID-19 Stratification Status (Table 3) and does not consider viral load as the study sought to investigate whether a one-time saliva sample during admission with ensuing microbiome analysis can predict disease severity. This severity score is based on the trend of the ACTT score, such as improvement or worsening, considering five assessment dates, and considering the treatment outcome (death or recovery). Five assessment dates were considered if the duration of hospitalization covered those dates: Baseline (day of admission), days 5, 15, and 30 after admission, and the day of discharge or expiration. The score was determined for each recruited patient after the outcome of the hospitalization was known.

**TABLE 3:** Classification of patients into two severity groups: critical or moderate.

ACTT code	Description
1	Not hospitalized, no limitations on activities
2	Not hospitalized, limitation on activities, and/or requiring home oxygen
3	Hospitalized, not requiring supplemental oxygen–no longer requires ongoing medical care
4	Hospitalized, not requiring supplemental oxygen–requiring ongoing medical care (COVID-19 related or otherwise)
5	Hospitalized, requiring supplemental oxygen
6	Hospitalized, on non-invasive ventilation or high flow oxygen devices
7	Hospitalized, on invasive mechanical ventilation or ECMO
8	Death during Study
**Severity group**	**Criteria**
Critical	Expired, or ACTT Status on Day 5 ≥ 5, and the patient deteriorated in any of the assessment dates 5, 15, or 30 days after admission
Moderate	Hospitalized but not categorized as critical

### Extraction and sequencing

The saliva samples were sequenced with two protocols: (1) shotgun DNA metagenomics (SDM) (Illumina), and (2) Illumina Respiratory Viral Oligo Panel (RVOP) sequencing (Illumina). DNA/RNA extraction was performed with the ZymoBIOMICS DNA/RNA Miniprep Kit. Quality control of SDM and RVOP libraries was performed using a Thermo Fisher Qubit 3.0 High Sensitivity DNA kit and an Agilent 2100 Bioanalyzer High Sensitivity DNA kit.

For the SDM, libraries were prepared, along with a ZymoBIOMICS Microbial Community DNA standard, using a Nextera XT DNA Library Prep kit following Illumina’s recommended guidelines (Illumina). Libraries were sequenced as paired-end, 2×150 bp, using a NextSeq 500 High-Output kit, with a 1% phi X sequencing control spike-in.

For the RVOP, libraries were prepared using an Illumina RNA Prep with Enrichment kit and enriched using the Respiratory Virus Oligos Panel v2 at 3-plex. Libraries were normalized by mass for three-plex enrichment but were not normalized by viral load as the viral copy number of each sample was unknown (Illumina). Libraries were sequenced as paired-end, 2×75 bp, using a NextSeq 500 Mid-Output kit, with a 1% phi X sequencing control spike-in.

### Bioinformatics analyses

Shotgun DNA metagenomics (SDM) reads were analyzed with 3 bioinformatics pipelines, Biobakery (Beghini et al., 2021), Kraken/Bracken (Wood et al., 2019), and Pathoscope (Hong et al., 2014). Biobakery KneadData was used for quality control on the SDM reads. RVOP reads were also analyzed with 3 bioinformatics pipelines: Basespace DRAGEN (Illumina), Kraken (Wood et al., 2019), and Nextflow’s nf-core/viralrecon (Ewels et al., 2020).

MetaPhlAn v3.0.7 with database vJan21 (Truong et al., 2015; Nousias and Montesanto, 2021) was run through Biobakery for profiling the composition of bacteria communities from the metagenomic data. Results presented here were run with a confidence parameter of –stat_q 0.1. Kraken and Bracken were run on the same sequence data for metagenomic profiling which includes DNA from viruses, fungi, parasites, and archaea. Results presented here were run with Kraken parameter –confidence 0.1.

HUMAnN2 (Franzosa et al., 2018) was run through Biobakery to profile the abundance of microbial metabolic pathways and other molecular functions from the metagenomics data. Tweediverse (Mallick et al., 2022) and MaAsLin2 (Mallick et al., 2021) were used for testing statistical associations with microbiome data, to compare community total abundances from MetaPhlAn or HUMAnN2 output with sample metadata. These approaches perform multiple tests with False Discovery Rate Correction using the Benjamini–Hochburg approach. MaAsLin2 uses linear models, and Tweedieverse uses Tweedie models to fit models and test hypotheses for microbial species.

MetagenomeSeq and DESeq2 tools were run within MicrobiomeAnalystR or the online MicrobiomeAnalyst (Chong et al., 2020). The software CARD’s Resistance Gene Identifier (RGI) 5.2.1 with the CARD database 3.2.0 (Alcock et al., 2020) was run with –include_wildcard and –aligner bowtie2 settings on the DNA shotgun metagenomic data to predict antimicrobial resistance phenotypes. CARD combines the antibiotic resistance ontology (ARO) with curated antimicrobial resistance (AMR) gene sequences and resistance-conferring mutations to provide an informatics framework for annotation and interpretation of resistomes (Alcock et al., 2020). Nextflow’s nf-core/viralrecon was run with default parameters on 83 RVOP samples that had enough viral reads. Basespace DRAGEN and Kraken were run on the RVOP reads to establish SARS-CoV-2 genome coverage.

The healthy subjects (controls) included from Armstrong et al. (2021) were health workers considered in good health and were negative for SARS-CoV-2 infection, based on concurrent qRT-PCR testing. We selected MetaPhlAn results from 5 subjects from the first sampling whose saliva did not undergo human depletion which we merged with our MetaPhlAn data and analyzed with MaAsLin using batch as random effect.

## Results

### Host clinical characteristics

A total of 120 patients were recruited for the CLAIRE study over a period of 1.5 months to provide a sample of saliva. With the exception of 8 patients whose saliva was sampled after day 3 upon hospital admission, saliva was sampled 3 days or less after admission to verify the hypothesis that an early saliva sample is predictive of disease progression. Four out of the 120 patients had a false positive COVID-19 test and 116 were confirmed to have COVID-19. Initially, the medical chart identified 4 false positive patients with a negative COVID-19 clinical diagnosis (see “Patient enrollment”). One false negative and one false positive patient were recategorized through virome analysis and clinical chart review, which resulted in 4 “False Positives” (FP) for subsequent analysis. The clinical and demographic characteristics of the patients are depicted in Table 1. The median age for the critical cohort was 60 (range 34–91) and for the moderate cohort 54 (range 19–86). When looking at COVID-19 risk factors other than age, based on past medical history, 43 patients had diabetes type II (12 critical, 28 moderate), 19 patients had chronic kidney disease, 19 patients had chronic obstructive pulmonary disease, 28 patients had or have cancer, and 43 patients had cardiovascular disease. A majority of the patients were Hispanic or Latino, with a 60%/52% breakdown of critical/moderate disease progression.

Correlation tests have been performed to identify collinearity among patient clinical information (metadata). To combine all results, −log(*p*-value) of features correlation was calculated with three different tests based on data types (Kruskal–Wallis, Spearman, Chi-Squared, Supplementary Figure 2). A table with *p*-values for the correlations can be found in Supplementary Table 1. Most significant pairwise correlations are obvious, e.g., race and ethnicity. Of most interest, is the significant correlation between the SARS-CoV-2 genome coverage and severity of disease, discussed in the next section.

Of the 12 patients who died, all of them (100%) received broad-spectrum sepsis antibiotics (BSA) during their hospitalization, resulting in a statistically significant association between sepsis treatment and mortality (*p* < 0.0001, Fisher exact test). 4 BSA sub-groups were selected based on the timing of the BSA treatment to understand its effects on the use and timing of saliva collection as a biomarker of disease progression. We therefore stratified the 120 patients into 2 BSA groups, each with 2 subgroups, and into two severity groups, with 95 (79%) subjects classified as being moderate and 25 (21%) subjects classified as being critical (see Table 2 and Table 3). See Supplementary Table 2 for only the 4 FPs’ outcomes.

Several clinical features were found to be significantly different between critical and moderate groups, among them C-reactive protein (CRP) values being lower for the moderate group when compared to the critical group (Mann–Whitney U test, *U* = 562.5, *p* = 0.030), which has been confirmed previously using proteomics (Rahnavard et al., 2022). Similarly, we observed significant associations for lactose dehydrogenase (LDH) being lower in the moderate group (Mann–Whitney U test, *U* = 470.5, *p* = 0.026), and the ratio of neutrophils to lymphocyte (NLR) also being lower in the moderate group (Mann–Whitney U test, *U* = 741.0, *p* = 0.037). All three (CRP, LDH, and NLR) are known COVID-19 biomarkers (Gogate et al., 2021).

### Disease severity association with SARS-CoV-2 genome coverage and variants

We found a significant association between percentage of SARS-CoV-2 genome coverage and severity of disease (Mann–Whitney U test, *U* = 638, *p* = 0.00168). A total of 8 samples with a low number of total reads for the RVOP sequencing (< 200,000) were removed from consideration because RVOP, which relies on an oligo-capture approach with very short paired 76 bp reads, has been found to be less reliable with low viral loads and low read counts (Lam et al., 2021). 83 samples had enough genome coverage to be categorized as a variant, of those, 61 were categorized as Delta 21J (Nextclade clade) with the most frequent lineage being AY.25.1, a predominant Canada and USA lineage. 1 patient variant was categorized as Mu 21H, a Colombia variant, and 1 patient as Gamma, V3 20J, a Brazilian/Manaus variant (see Supplementary Table 3). The 2 patients with non-Delta variants had moderate disease progression.

### Microbiome results

**FIGURE 1:**
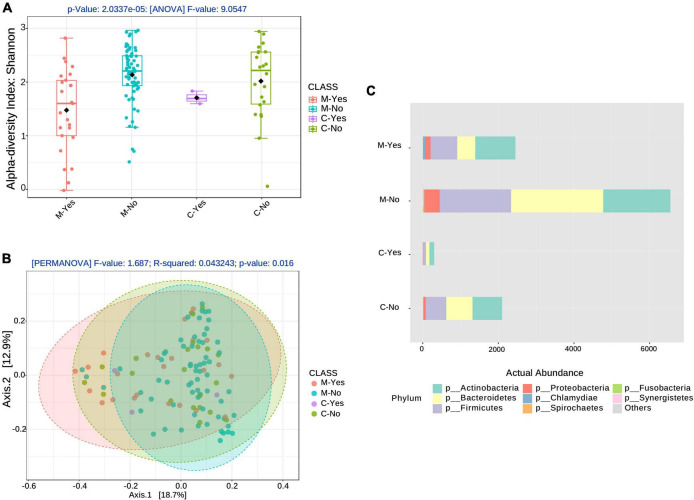
Alpha and Beta diversity associated with broad-spectrum sepsis antibiotics (BSA). **(A)** Alpha diversity boxplots for the COVID-19 groups, moderate (M) and critical (C) COVID-19 groups stratified by BSA timing [BSA before or same day as saliva sampling: Yes (Y), No (N)]. **(B)** Beta diversity with COVID-19 groups, as before, stratified by BSA timing. **(C)** Phyla abundance plot.

Shotgun metagenomics generated an average of 4 million reads/sample. Quality control with the Biobakery KneadData tool removed an average of 1.5 million low-quality reads/sample, filtering out an average of 2 million human reads/sample using the hg38 human genome as a host reference (Supplementary Table 4). The Biobakery MetaPhlAn tool identified an average of 700,000 bacterial reads/sample and 332 species from which diversity estimates were made (Figure 1). The Kraken/Bracken combination of tools (confidence level 0.1) identified 2264 bacterial species, 123 DNA virus species, and 60 fungi and parasite species from the 120 CLAIRE patient oral samples.

Figures 1A, B show alpha- and beta-diversity at the species level after stratification into 4 groups: moderate with early BSA treatment (M-Yes), Moderate with no early BSA treatment (M-No), Critical with early BSA treatment (C-Yes), Critical with no early BSA treatment (C-No), where “early” was defined as the treatment being initiated before or on the same day as saliva sampling. Alpha-diversity analysis, as shown in Figure 1A, returned a significant result (*p* = 2.0337*e*−5), as did the beta-diversity analysis (*p* = 0.016), shown in Figure 1B. The taxa abundance plot in Figure 1C shows the abundance of bacteria at the phylum level for the stratified groups for a high-level view of the microbiome composition differences among the groups. When including data from the false positives and 5 healthy subjects (controls) from the study by Armstrong et al. (2021) (see “Materials and methods” section) with the CLAIRE patient data, alpha- and beta-diversity analyses were significant at even lower *p*-values (Supplementary Figure 3). Beta-diversity for the 4 BSA categories was also significant at the genus level (*p* < 0.001) (Supplementary Figure 4).

**FIGURE 2:**
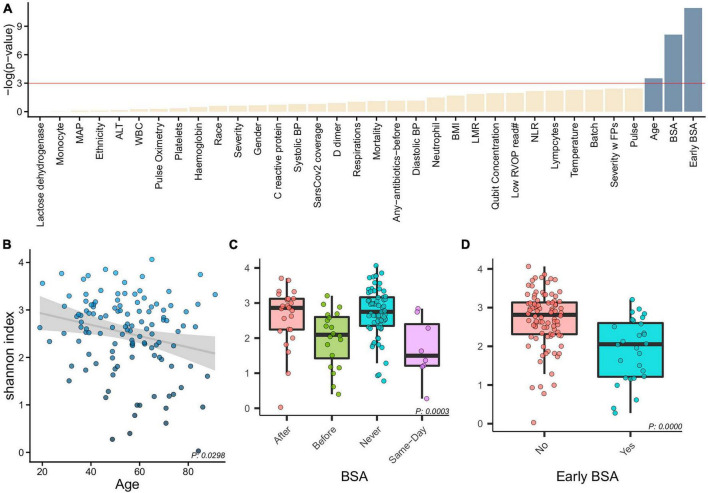
Alpha diversity and clinical characteristics. **(A)** Associations between alpha diversity and sample clinical information (*x*-axis) and statistical significance (–log(*p*-value)–*y*-axis) with a horizontal red line showing the –log(0.05) cutoff for significance. With age, BSA and early BSA showing significance, we show alpha diversity against the categorizations of each of these clinical variables, namely, **(B)** Age, **(C)** BSA, and **(D)** early BSA.

Alpha diversity associations with clinical biomarkers are shown in Figure 2. The most significant associations were with age (*p* = 0.0298, Figure 2B), with the 4 BSA categories (*p* = 0.0003, Figure 2C), and the 2 Early BSA groups (*p* < 0.0001, Figure 2D). As expected, BSAs disrupted the bacterial saliva microbiome with a significant reduction in alpha-diversity for the Early BSA group. The difference is slightly more marked at the genus level (*p*-value = 1.8504e-05) (Supplementary Figure 5).

#### Healthy subjects and false positives comparison

**FIGURE 3:**
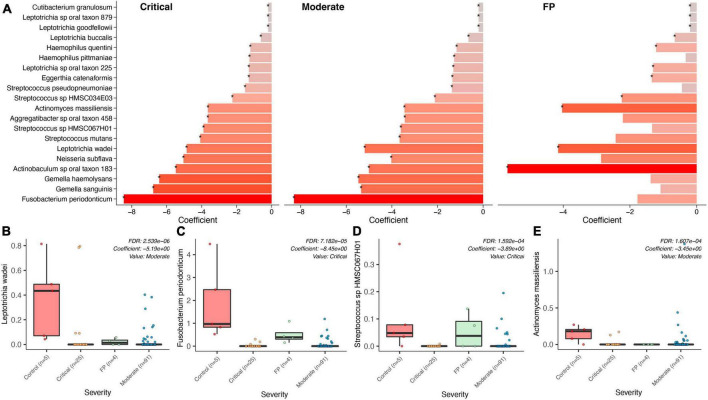
Bacterial associations across COVID-19 groups, FP, Controls. **(A)** Top bacteria found in a comparison of the bacterial microbiome composition between the two COVID-19 groups (severe, moderate), the False Positives (FP), and the control group (healthy individuals) using control as reference. **(B–E)** Show specific significance tests for those bacterial species found to be significantly different between controls and our three case groups.

We compared data from 5 healthy subjects (controls) from the study by Armstrong et al. (2021) (see “Materials and methods” section) with the CLAIRE patients (Figures 3A–E) and found *Fusobacterium periodonticum* was among the most reduced species in COVID-19 critical patients with respect to controls (Coefficient: -8.45, *p* = 1.069e-06, FDR = 7.182e-05), see Figure 3C. Other significantly reduced species in the two COVID-19 groups were *Leptotrichia wadei* (Coefficient: −5.19, *p* = 1.092E-08, FDR = 2.539E-06), *Actinomyces massiliensis* (Coefficient: −3.45, *p* = 3.666E-06, FDR = 1.607E-04), *Gemella haemolysans* (Coefficient: −6.43, *p* = 3.097E-06 FDR = 1.487E-04), and *Streptococcus* sp HMSC067H01 (Coefficient: −3.89, *p* = 3.475E-06 FDR = 1.592E-04) (Supplementary Table 5). Genus level results are provided in Supplementary Table 6.

A significant association distinguishing the bacteria in the saliva of FP patients with that in COVID-19 patients was found. As shown in Supplementary Table 7, *Prevotella shahii* (*p* = 1.84E-08, FDR = 5.86E-06), *Fusobacterium periodonticum* (*p* = 8.70E-08, FDR = 1.24E-05) and *Leptotrichia* sp oral taxon_215 (*p* = 1.17E-07, FDR = 1.24E-05) resulted as the most significantly reduced species in COVID-19 patients with respect to the cluster of FPs.

### Disease severity associations with microbiome composition

This section explores disease severity independent of BSA drugs. Our analyses reveal significant differences in the non-bacterial microbiome and its potential as a biomarker of disease progression, presented below, while the bacterial microbiome did not show significant differences. The “No early BSA” section, however, presents significant differences between moderate and critical groups.

#### Disease severity, DNA viruses, fungi, and parasites

DNA viruses, fungi, and parasites were studied together using the Bracken data as there were fewer species among fungi/parasites: 60, DNA virus: 123, than bacterial species: 2272. Beta diversity of DNA virus, fungi/parasites was significantly different for the moderate and critical groups only at the phylum level (PERMANOVA, *p* < 0.041). DNA virus, fungi/parasites alpha diversity was borderline significant at the family level and lower for the critical group (*p* < 0.043) (Supplementary Figure 6).

**FIGURE 4:**
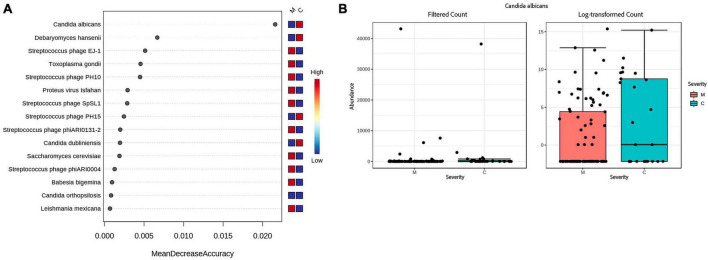
Accuracy of predicting disease severity by microbial species. **(A)** Random forest analysis of the decrease in accuracy (*x*-axis) of predicting disease severity with the removal of individual species (*y*-axis). A higher mean decrease accuracy value indicates the importance of that species in predicting severity of disease (moderate vs. critical). **(B)** Because *Candida albicans* was the most important species with respect to disease prediction, we further examined this taxon via a Box and Whisker plot to show individual data points.

When looking at severity of disease, 2 fungi, *Candida dubliniensis* (*p* = 0.000274, FDR = 0.001369), *Candida albicans* (*p* = 0.002891, FRD = 0.0096352), were found by all bioinformatics tools to be significantly elevated in the critically ill group. Proton pump inhibitors (PPI) are a risk factor for the identification of *Candida* but medical records show the same percentage of PPI medication, 32%, in both severity groups. *Streptococcus* phage EJ-1 (*p* = 4.20E-07, FDR = 4.20E-06), *Streptococcus* phage SpSL1 (*p* = 5.44E-06, FDR = 3.63E-05), *Streptococcus* phage phiARI0131-2 (*p* = 0.005, FDR = 0.014305), and *Streptococcus* phage PH10 (*p* = 0.007, FDR = 0.018306), were found to be significantly elevated in the moderate group (see Supplementary Table 8). A random forest classification on the Bracken data finds *Candida albicans* as the top feature contributing to classification accuracy (Mean Decrease Accuracy of 0.025) between moderate and critical patients (see Figures 4A, B). The out of bag (OOB) error rate was 16.7% with only 1/95 moderate patients misclassified, and 6/25 critical patients correctly classified.

### BSA treatment effects on microbiome composition

#### BSA and bacteria

We found no significant association between SARS-CoV-2 genome coverage and the timing of BSAs for sepsis treatment (Mann–Whitney U test, *U* = 938.5, *p*-value = 0.2187) indicating that COVID-19 viral load is not correlated with sepsis. A significant association was found between patients with active cancer and broad-spectrum antibiotic treatment for sepsis early upon admission (Fisher exact test, *p* < 0.015), indicating that immune suppression correlates with sepsis.

MaAsLin finds 72 significant differences between the BSA groups, as shown in Supplementary Table 9. The most significantly reduced bacteria in the Early BSA group was *Prevotella pallens* (Coefficient: −2.834, *p* = 4.58E-05, FDR = 0.007279), the next significantly reduced species was *Alloprevotella tannerae*, with much higher *p*-value (Coefficient: −3.131, *p* = 0.000997, FDR = 0.05283). Multiple bacteria were found to be significantly associated with the 4 BSA categories at the MaAsLin threshold of *q*-val < 0.25 when analyzing the MetaPhlAn data. *Prevotella pallens* was again significantly reduced in the “Before” group (Coefficient: −3.195, *p* = 9.30E-05, FDR = 0.044356). The next significant bacteria after *Prevotella pallens* had a much higher *p*-value and was *Actinomyces* sp oral taxon 1 (*p* = 0.001892, FDR = 0.23859). The 5 significant genera in common among the results by the three different tools (DESeq2, MetagenomeSeq, MaAsLin) and the two datasets (MetaPhlAn, Bracken) were *Selenomonas*, *Prevotella*, *Gemella*, *Fusobacterium*, and *Haemophilus* (DESeq2 FDR < 0.05).

#### BSA and DNA viruses, fungi, and parasites

Alpha diversity was not significantly different between the BSA groups when studying fungi and parasite species indicating being less affected by BSAs. Alpha diversity for DNA virus species was significantly lower for the DNA virus species for the Early BSA group (*p* < 7.43e-5). See Supplementary Figure 7.

#### BSA and antimicrobial resistance

**FIGURE 5:**
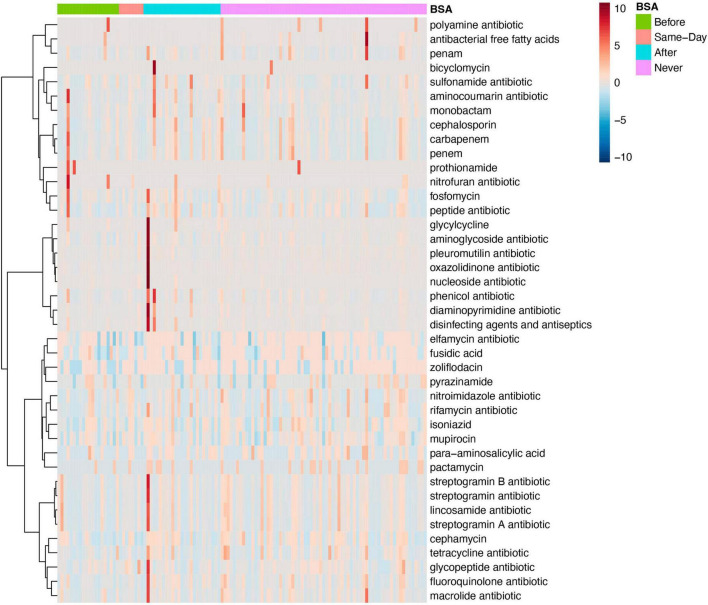
Antimicrobial resistance for specific antibiotics by broad-spectrum sepsis antibiotic (BSA) timing. Heatmap of samples with antimicrobial resistance to single drug classes grouped by BSA timing.

Both MaAsLin and MetagenomeSeq find tetracycline antibiotic resistance to be significantly lower in the Group with “Early BSA” (MetagenomeSeq, *p* = 0.0037681, FDR = 0.030898). As the same drug resistance can appear in genes of numerous different bacterial or pathogen species, the analysis focuses on the drug classes. When looking at mixed AMR drug classes, MaAsLin and MetagenomeSeq found 2 different mixed drug classes that both contain resistance to fluoroquinolone and were significantly lower in the “Early BSA” group (MetagenomeSeq, *p* = 1.57E-06, FDR = 0.000101). In both cases, the mixed drug class also contains the tetracycline antibiotic (Supplementary Figure 8). Tetracycline (e.g., doxycycline hyclate) was given to 6 patients after saliva sampling, 4 of whom received BSAs, and neither had high resistance to tetracycline. A frequently used fluoroquinolone agent, ciprofloxacin, was not found in the medical records for the days of hospitalization, but another fluoroquinolone, levofloxacin, was administered to 8 patients, 5 of whom also received BSAs, with 1 having a somewhat elevated resistance to fluoroquinolone but whose disease progression was moderate. Patients P8 and P29 are both patients with elevated numbers of drug resistance bacteria not present in other patients (see dark long red lines in the heatmap in Figure 5). Both were prescribed BSAs after saliva sampling (“After” Group) and developed severe COVID-19 symptoms (“Critical” Group), and patient P8 expired. See the section “No early BSA and antimicrobial resistance”.

#### BSA and bacterial functional annotation

**FIGURE 6:**
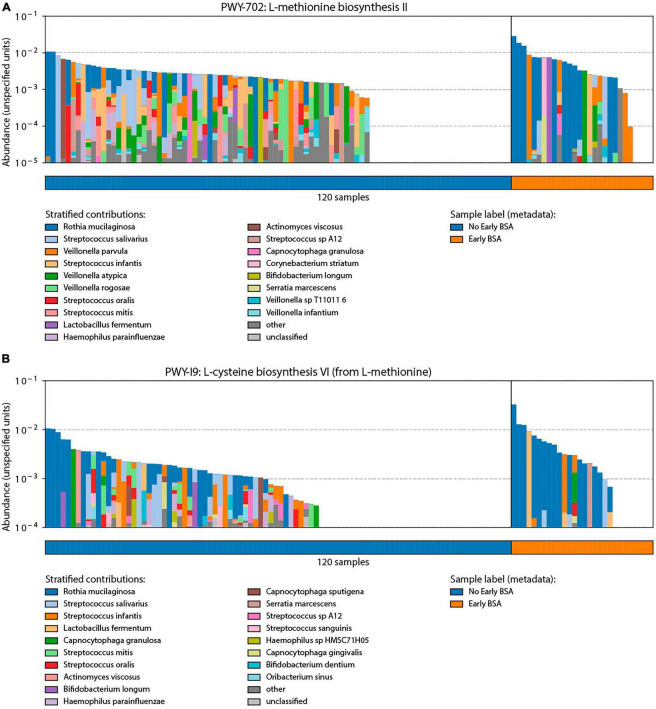
Analysis of broad-spectrum sepsis antibiotic (BSA) classification by bacterial functional group. Significant pathways identified through bacterial functional analysis relative to BSA status with *y*-axis length or area of each colored segment showing the relative abundance of a taxonomic group in the given pathway: **(A)** L-methionine biosynthesis II pathway that was preferentially enriched in the Early BSA samples. **(B)** L-cysteine biosynthesis VI pathways were also enriched in the Early BSA samples.

Bacterial functional annotation data (generated with Humann2) were analyzed by MaAsLin for the BSA groups. Two pathways were significantly elevated in the group that started with BSA early, before saliva sampling (*p*-value < 0.00015, FDR < 0.123): (1) PWY-702: L-methionine biosynthesis II: *Rothia mucilaginosa*, and (2) PWY-I9: L-cysteine biosynthesis VI (from L-methionine): *Rothia mucilaginosa*. As shown in Figure 6, the two pathways were not active in a larger proportion of samples in the No early BSA group (empty area in chart) while a majority of the samples in the Early BSA group showed the two pathways active with a higher number of bacteria associated with the pathways.

### Absence of BSA treatment and its effect on microbiome composition and association with severity of disease

Patients who were not treated with BSAs before saliva sampling were studied separately to find patterns of disease progression not affected by these antibiotics (see Table 2). Among the “No early BSA” patients (*n* = 92), “After” patients, who developed sepsis after sampling (*n* = 25), were compared to “Never” patients, who never developed sepsis (*n* = 67).

#### No early BSA, disease severity, and bacteria

Unlike results of analysis including all patients, the “No early BSA” analysis of the Bracken data with MaAsLin finds bacteria that are significantly more elevated in the “Moderate” group: *Streptococcus pneumoniae*, *Streptococcus pseudopneumoniae*, and *Campylobacter concisus* (*p* < 0.00043, FDR < 0.061). MetagenomeSeq finds *Staphylococcus aureus* significantly more elevated in the Critical group (*p* = 0.000103, FDR = 0.027811). Medical records show *Staphylococcus aureus* was found in blood culture results of 3 patients. At the genus level, in the MaAsLin analysis, some bacteria are significantly more elevated (*Streptococcus*, *Gemella*, *Lachnoanaerobaculum*) or significantly more reduced (*Bacillus*) in the “Moderate” group (*p*-value < 0.0015, *q*-val = 0.055).

#### No early BSA, disease severity, and antimicrobial resistance

When considering only the “No early BSA” patients, MetagenomeSeq finds AMR drug multiclasses significantly elevated in the moderate group and containing drugs that were found to be significant in the earlier analysis of the 2 main BSA groups: “macrolide antibiotic; fluoroquinolone antibiotic; tetracycline antibiotic; phenicol antibiotic; disinfecting agents and antiseptics” (*p* = 4.33E-16, FDR = 4.29E-14). See “Discussion” section on AMR.

#### No early BSA, disease severity, DNA virus, fungi and parasites

The results for the “No early BSA” group when studying DNA viruses, fungi, and parasites are comparable to results analyzing all patients, possibly because these components of the microbiome are not as affected by the BSA drugs (Supplementary Table 8).

## Discussion and conclusion

In our CLAIRE study, the saliva microbiome was analyzed as a possible biomarker predictive of disease progression in infectious respiratory diseases, specifically COVID-19 and sepsis BSA treatment, to aid in the triage of patients in a hospital setting. We found that bacterial microbiome differences between the groups were mainly due to BSA treatment rather than the COVID-19 infection. Random forest analysis revealed a surprising link between *Candida albicans* and disease severity, suggesting a previously unrecognized role for this fungus as a biomarker for disease progression. Our study found *Staphylococcus aureus* significantly more elevated in the Critical group of patients who did not receive BSA before saliva sampling. Another relevant finding is the significantly elevated number of *Streptococcus* phage in the moderate group, and while *Streptococcus* phages are one of the least studied bacteriophages, recent publications suggest studying phages as “living antibiotics”(Schooley and Strathdee, 2020; Mehmood Khan et al., 2023).

To date, no studies have completely addressed saliva microbiome profiling, including fungal, parasitic, and viral components in COVID-19 and sepsis treatment and have studied specimens other than saliva, such as oropharynx, nasopharyngeal, oral rinses, throat, or oral swabs (Lebba et al., 2020; Ma et al., 2021; Miller et al., 2021; Nardelli et al., 2021; Soffritti et al., 2021; Li et al., 2023). Our study confirmed the impact of antibiotics in reducing the diversity of the bacterial saliva microbiome compared to the viral, parasite, and fungal saliva microbiome, which contained possible biomarkers of disease progression. Our study reaches similar conclusions to a meta-analysis study by Alshaikh et al. (2022), providing evidence for the need to use narrow-spectrum agents that specifically target pathogenic bacteria supporting better antimicrobial stewardship (AMS) practices using a laboratory-confirmed diagnosis of coinfection instead of the systematic use of broad-spectrum antibiotics. Di Pilato and Giacobbe (2023), noted that “the overall prevalence of bacterial infections in hospitalized COVID-19 patients remains low (4–6%) a striking contrast with the high prevalence of antibiotic use reported by the same population (60–100% in most studies)”, recommending AMS interventions that reduce antibiotic resistance (Di Pilato and Giacobbe, 2023). Nandi et al. (2023) found that antibiotic sales were positively associated with COVID-19 cases globally during 2020-2022 and pointed out the ongoing necessity to enhance better antibiotic stewardship practices in the context of COVID-19. Our findings suggest there is a need for research looking into the possible protective or deleterious effect of specific bacteria on the development of an infection and into the effect of timing of BSAs on whether the bacterial microbiome or the non-bacterial microbiome of a saliva sample should be considered as a candidate biomarker of infectious disease severity. Based on Yonkus et al. (2022) findings, nanopore sequencing, which takes 8 h compared to 98 h to return rapid microbial profiling results, has great potential to improve antibiotic stewardship. This method should be accompanied by a prescription of narrow-spectrum antibiotics, once scientific and clinical research align their efforts to develop such agents (Alm and Lahiri, 2020; Datta, 2023).

### Associations with severity of disease

Oral COVID-19 microbiome studies have not looked into the effect of antibiotics treatment and have mostly focused on bacteria (Ma et al., 2021; Miller et al., 2021; Nardelli et al., 2021; Soffritti et al., 2021; Li et al., 2023), except for an Ion Gene Studio S5 sequencing study on oral rinse samples that looked at non-bacterial microbiome components (Lebba et al., 2020). Our study expands the focus beyond bacteria in a combined analysis of BSA and sepsis and finds significant associations between DNA viruses, fungi, and parasites and severity of infectious disease (both sepsis and COVID-19). Similar to what was observed in larger series, the majority of patients with COVID-19 (79% or 95 patients) responded to the standard management and were classified as moderate (moderate severity group), while a smaller proportion (21% or 25 patients) became critically ill or died (critical severity group) (see Table 3).

#### DNA viruses, parasites, and fungi

As all patients received antibiotics, the bacterial saliva microbiome did not reveal any statistically significant difference between the moderate and critical severity groups, however, the DNA viral, parasitic, and fungal components of the microbiome, not targeted by the antibiotics, exhibited some significant differences. Specifically, *Candida albicans* emerged as a predictive biomarker of disease severity based on random forest analysis. Immunomodulatory therapy, which became part of the standard therapy for COVID-19, is known to influence the fungal burden in the microbiome. Recent studies have described an increase in deaths from fungal infections during the COVID-19 pandemic (Mina et al., 2022; Gold et al., 2023). A history of proton pump inhibitors (PPI) is a risk factor for the identification of *Candida* (Mojazi Amiri et al., 2012), but PPIs were prescribed proportionally at 32% in both severity groups.

Along with *Candida albicans*, *Candida dubliniensis* was also significantly elevated in the critical group, and several *Streptococcus* phage species were elevated in the moderate group, among them, *Streptococcus* phage EJ-1 and phage PH10, which Soffritti et al. (2021) found increased in COVID-19 patients compared to controls when studying oral rinse samples. *Streptococcus* sp HMSC067H01, an unclassified *Streptococcus* species, was found to be significantly reduced in COVID-19 patients and higher in controls (Soffritti et al., 2021), which could explain the elevated number of *Streptococcus* phage in moderate disease progression. The BSAs used in this study are currently being studied for phage combination therapies (Save et al., 2022; Osman et al., 2023).

#### No-early BSA group

To investigate whether it is possible to identify biomarkers of disease progression when BSAs were not administered, we excluded patients treated with BSAs before saliva sampling and looked at the “No early BSA” patients (*n* = 92) (see Table 2). At the genus level, *Streptococcus*, *Gemella*, and *Lachnoanaerobaculum* are significantly more elevated and *Bacillus* is significantly more reduced in the “Moderate” group. Interestingly, the results for DNA viruses, parasites, and fungi, when considering only “No early BSA” patients, were comparable to results considering all patients. This provides evidence that unlike bacteria, DNA viruses, parasites, and fungi are not affected by BSAs and can be used as a biomarker of disease progression regardless of whether BSAs were administered.

### Sepsis and broad-spectrum sepsis antibiotics

#### Sepsis

We investigated whether sepsis could be caused by a more severe COVID-19 infection. A Mann–Whitney U test did not support the second hypothesis, with the SARS-CoV-2 genome coverage between the early or not early BSA groups not being significantly different. This indicates sepsis is associated with other conditions than COVID-19, such as possibly a weaker immune system, which is corroborated by a Fisher exact test finding a significant association between the 2 sepsis groups and patients with active cancer. We saw a significant reduction of *Prevotella pallens* (Pp) in the early BSA group, while Miller et al. (2021) saw a significant enrichment of Pp in COVID-19 patients in their 16S rRNA study but did not consider antibiotics use. In our study, 96% of COVID-19 patients who did not receive BSAs before sampling did not exhibit a critical disease progression and had a statistically significant higher abundance of Pp. It is possible that Pp is enriched in COVID-19 and is also especially susceptible to BSAs. Microorganisms identified from blood culture (sampled on different dates than saliva sampling) in two or more sepsis-treated patients were *Escherichia coli* (5), *Candida albicans* (4), *Staphylococcus aureus* (3), *Enterococcus faecalis* (3), *Pseudomonas aeruginosa* (2) (number of patients shown in parenthesis). In our saliva microbiome analysis, *Candida albicans* and *Staphylococcus aureus* were found to be significantly elevated in critical patients. More research is needed to study the potential of *Staphylococcus aureus* to be predictive of sepsis and potential linkages between the blood microbiome and the saliva microbiome, which would require simultaneous sampling of blood and saliva.

#### Antimicrobial resistance (AMR)

Significant AMR differences were found between the 2 BSA groups but not between the two COVID-19 disease severity groups. Tetracycline antibiotic resistance (TAR) and fluoroquinolone antibiotic resistance (FAR) were found to be significantly lower in the “Early BSA” group. This could indicate that either BSAs were effective at killing the TAR/FAR bacteria, which is a more likely explanation as they target highly resistant organisms in nosocomial infections, or that the patients with sepsis had fewer resistant bacteria, which is unlikely but supported by two findings. First, the stratification into 4 BSA groups in which the group that developed sepsis after saliva sampling had a lower abundance of TAR bacteria, the group that never developed sepsis had the largest abundance of TAR bacteria (see Supplementary Figure 8), and second, an AMR multiclass containing tetracycline and fluoroquinolone resistance was also significantly elevated in the moderate group when looking at the no early BSA subset of patients. Although Tetracycline (e.g., doxycycline hyclate) and fluoroquinolone (e.g., levofloxacin) were given to some patients (less than 10), we could not assess whether the resistance pattern affected disease progression as most also took BSAs, did not show high resistance to those agents, and had a moderate disease progression. In the “No early BSA” group, multiclass AMRs were significantly elevated in the moderate subgroup and contained AMR to drugs mentioned above but also to disinfecting agents and antiseptics. The heightened use of disinfectants during COVID-19 may have led to AMR (van Dijk and Verbrugh, 2022). Two patients with the highest numbers of diverse drug resistance bacteria not present in other patients (see heatmap in Figure 5) were in critical condition and one died, but a larger sample size is needed to show AMR affected their disease progression.

### Bacterial saliva microbiome diversity and composition in COVID-19 patients

#### *Fusobacterium periodonticum* (Fp)

*Fusobacterium periodonticum* (Fp) was among the most significantly reduced species in the COVID-19 patients as compared to the false positives, in line with a nasopharyngeal microbiome case/control study (Nardelli et al., 2021), with similar trends observed between the salivary and nasopharyngeal microbiome (Kim et al., 2023). Nardelli et al. (2021) reported that Fp can perform surface sialylation and that some sialic acid residues on the cell surface could work as additional S protein of SARS-CoV-2 receptors.

#### Species richness

Our study noted a significant diminution in bacterial species richness in COVID-19 patients, which was also observed in Lebba et al. (2020), a 16S rRNA microbiome study from naso/oral-pharyngeal swabs (Lebba et al., 2020). In that study, unlike ours, patients did not take antibiotics or probiotics before sampling. We observed that BSA significantly reduced bacterial alpha diversity reinforcing the observations that BSAs have a strong effect on the saliva microbiome (see Figure 1). We also found a significant decrease in species diversity with growing age, which is confirmed by other oral microbiome studies (Huang et al., 2020; Willis et al., 2022).

#### Specific group differences

We found a significant reduction in bacterial abundance in the COVID-19 patients, with the top reduced genera being *Fusobacterium*, *Kingella*, *Actinobaculum*, *Gemella*, *Leptotrichia*, *Cardiobacterium*, *Aggregatibacter*, *Parvimonas*, *Haemophilus*, *Campylobacter*, *Lachnoanaerobaculum*, and *Neisseria*, in that order. Of those, the main bacterial genera in the normal oral cavity include *Fusobacterium*, *Leptotrichia*, and *Neisseria* (Li et al., 2014). Soffritti et al. (2021) found many species of periodontopathogenic bacteria that were significantly increased in COVID-19 oral rinse samples compared to control subjects. In Ma et al. (2021), significantly higher levels of *Veillonella* were found in both COVID-19 and flu patients than in the control group when studying oropharynx swab specimens. Wu et al. (2021) found through 16S rRNA sequencing on throat swab samples that periodontitis-correlated taxa, including species *R. mucilaginosa* (Rm) were elevated in COVID-19 compared to controls. In contrast, Miller et al. (2021) found through 16S rRNA saliva microbiome analysis that Rm was enriched in control patients. In our study, Rm was significantly elevated in patients receiving BSAs the same day of sampling, suggesting that perhaps a more severe early infection, or sepsis, rather than COVID-19, may cause some of these bacteria to become more abundant. Wu et al. (2021) also found that species *H. parainfluenzae* and *N. subflava* were enriched in the oral microbiome of COVID-19 patients, but our study found a significant reduction of those bacteria in COVID-19 and the early BSA groups. Among the three “red complex” periodontal pathogens (Abdulkareem et al., 2023), only *P. gingivalis* was among the top 33 significantly reduced bacteria in the early BSA groups in our study (Supplementary Table 8).

Two of the studies cited above did not mention antibiotic usage, while Wu et al. (2021) reported that 67.9% of patients in their study were treated with antibiotics to prevent potential secondary bacterial co-infections and that they adjusted for it. Antibiotic usage may have had a confounding effect in all those studies, even for the Wu et al. (2021) study after adjustment, and the difference in COVID-19 variants, study population, sequencing method, or sample method has to be considered when comparing results. Our study corroborated some of Wu et al. (2021) findings with *Neisseria*, *Corynebacterium*, and *Aggregatibacter* being significantly lower in the early BSA group and significantly decreased in the COVID-19 group.

#### Functional pathways

Two L-Methionine pathways were significantly elevated in the early BSA group. A recent hypothesis postulates that L-Methionine may modulate the assembly of SARS-CoV-2 by interfering with the mechanism of RNA polymerase (Benavides, 2022). SARS-CoV-2 could therefore benefit from the enrichment of the two pathways. Some bacteria, such as *Rothia mucilaginosa* (Rm), can synthesize methionine. As pointed out above, different studies found contradicting abundance patterns for Rm (Miller et al., 2021; Wu et al., 2021). In our study, Rm was significantly elevated in patients who started BSA on the same day of sampling. Bacteria involved in the L-Methionine biosynthesis pathway may have contributed to the sepsis symptoms. As most patients in the early BSA group did not develop a critical disease progression, BSAs may have eliminated these bacteria, disrupting the L-Methionine pathways that could no longer be exploited by SARS-CoV-2. The role of Rm may need to be further investigated.

## Data availability statement

The data presented in this study is available at https://www.ncbi.nlm.nih.gov/bioproject/?term=PRJNA883997.

## Ethics statement

The studies involving humans were approved by the University of Miami, IRB ID: 20200724. The studies were conducted in accordance with the local legislation and institutional requirements. The participants provided their written informed consent to participate in this study.

## Author contributions

KF: Conceptualization, Data curation, Formal analysis, Investigation, Validation, Visualization, Writing – original draft, Writing – review & editing. CR: Investigation, Methodology, Resources, Validation, Writing – review & editing. AR: Formal analysis, Investigation, Methodology, Software, Visualization, Writing – review & editing. KC: Formal analysis, Investigation, Methodology, Resources, Supervision, Writing – review & editing. JC: Conceptualization, Investigation, Methodology, Project administration, Writing – review & editing.
