# Supplementary material for: Hospital antimicrobial stewardship: profiling the oral microbiome after exposure to COVID-19 and antibiotics
Source: Front Microbiol. 2024 Feb 27;15:1346762. doi: 10.3389/fmicb.2024.1346762 (PMC10927822; doi:10.3389/fmicb.2024.1346762)
Supplement: Supplementary file 1 [file Data_Sheet_1.pdf]

SUPPLEMENTARY MATERIALS FOR

Hospital Antimicrobial Stewardship: Profiling the Oral Microbiome After Exposure to COVID and Antibiotics

Patricia Buendia[1*], Krystal Fernandez[1], Castle Raley[2], Ali Rahnavard[3], Keith A. Crandall[2,3], Jose Guillermo Castro[4]

[1]Lifetime Omics, Miami, FL 33133

[2]The GWU Genomics Core, Milken Institute School of Public Health, The George Washington University, Washington, DC 20052

[3]Computational Biology Institute, Department of Biostatistics and Bioinformatics, Milken Institute School of Public Health, The George Washington University, Washington, DC 20052

[4]Division of Infectious Diseases, Leonard M. Miller School of Medicine, University of Miami, Miami, FL 33136

This PDF file includes:

Figure S1. Study Workflow

Figure S2. Collinearity between metadata variables including clinical and patient characteristics.

Figure S3. Alpha and Beta diversity associated with Broad-Spectrum Sepsis Antibiotics (BSA) for all patients and 5 healthy subjects

Figure S4. Bacterial Microbiome Beta Diversity at the Genus Level

Figure S5. Bacterial Microbiome Alpha Diversity at the Genus Level

Figure S6. Alpha Diversity at the Family level by Disease Severity groups of DNA Viruses, Fungi and Parasites

Figure S7. Species Alpha Diversity by BSA groups of **(a)** Fungi and Parasites **(b)** DNA Viruses

Figure S8. Maaslin Boxplots showing antimicrobial resistance differences between BSA groups

<u>Other supplementary materials for this manuscript include the following:</u>

Table S1. P-values for Correlations shown in Figure S2

MetadataCorrelation_Pvalue.xlsx

Table S2. Outcome for 4 False Positives

Supp-Table-4-false-positives-outcome-antibiotics.xlsx

Table S3. Nextflow nf-core/viralrecon variant analysis

Coverage-variants.xlsx

Table S4. Microbiome read counts by Biobakery (BB) and Kraken (K) and Respiratory Virome Sequencing (RVOP) read counts and genome coverage

Kraken-Biobakery-Read-Stats-Supp-Material-deid.xlsx

Table S5. Severity and Controls at Species Level - Significantly reduced bacteria at the species level in COVID-19 compared to healthy subjects (controls)

AR_Maaslin2_Species_significant_results.xlsx

Table S6. Severity and Controls at Genus Level- Significantly reduced bacteria at the genus level in COVID-19 compared to healthy subjects (controls)

Masslin_Severity_Healthy_Armstrong_Genus_Level_Significant_Results.xlsx

Table S7. Severity and FPs - Significantly reduced bacterial species in COVID-19 patients as compared to false positives

Maaslin_Severity_4FPs_significant_results.xlsx

Table S8. DNA Virus, fungi, and parasites - Species/Genus - Significant associations between severity of disease and DNA Virus, fungi, and parasites

DNA-Virus-Fungi-Parasites-Severity.xlsx

Table S9. BSA and Bacteria - Significantly reduced bacterial species in patients receiving broad-spectrum sepsis antibiotics

Maaslin-sepsis_bef_significant_results.xlsx

Table S10. Species Abundance Table Kraken/Bracken

bracken_c0.1_combined_subshell2.S-reid.txt

Table S11. Genus Abundance Table Kraken/Bracken

bracken_c0.1_combined_subshell2.G-reid.txt

Table S12. Abundance Table Metaphlan

claire_metaphlan_concat_01242022_merged_metaphlan_with_Armstrong21_reid.txt

Figures

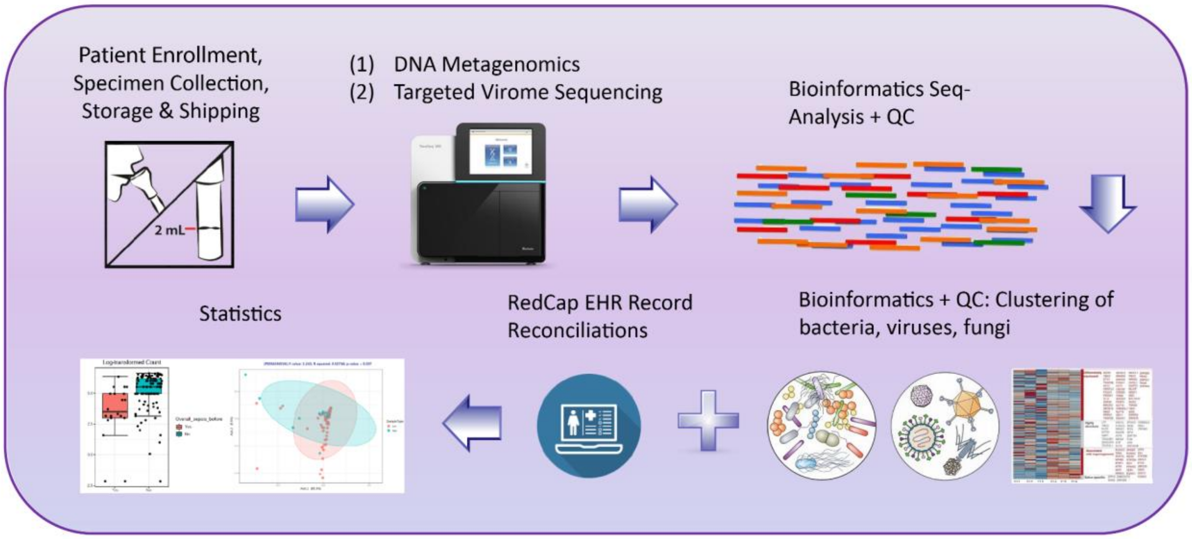

Figure S1. Study Workflow

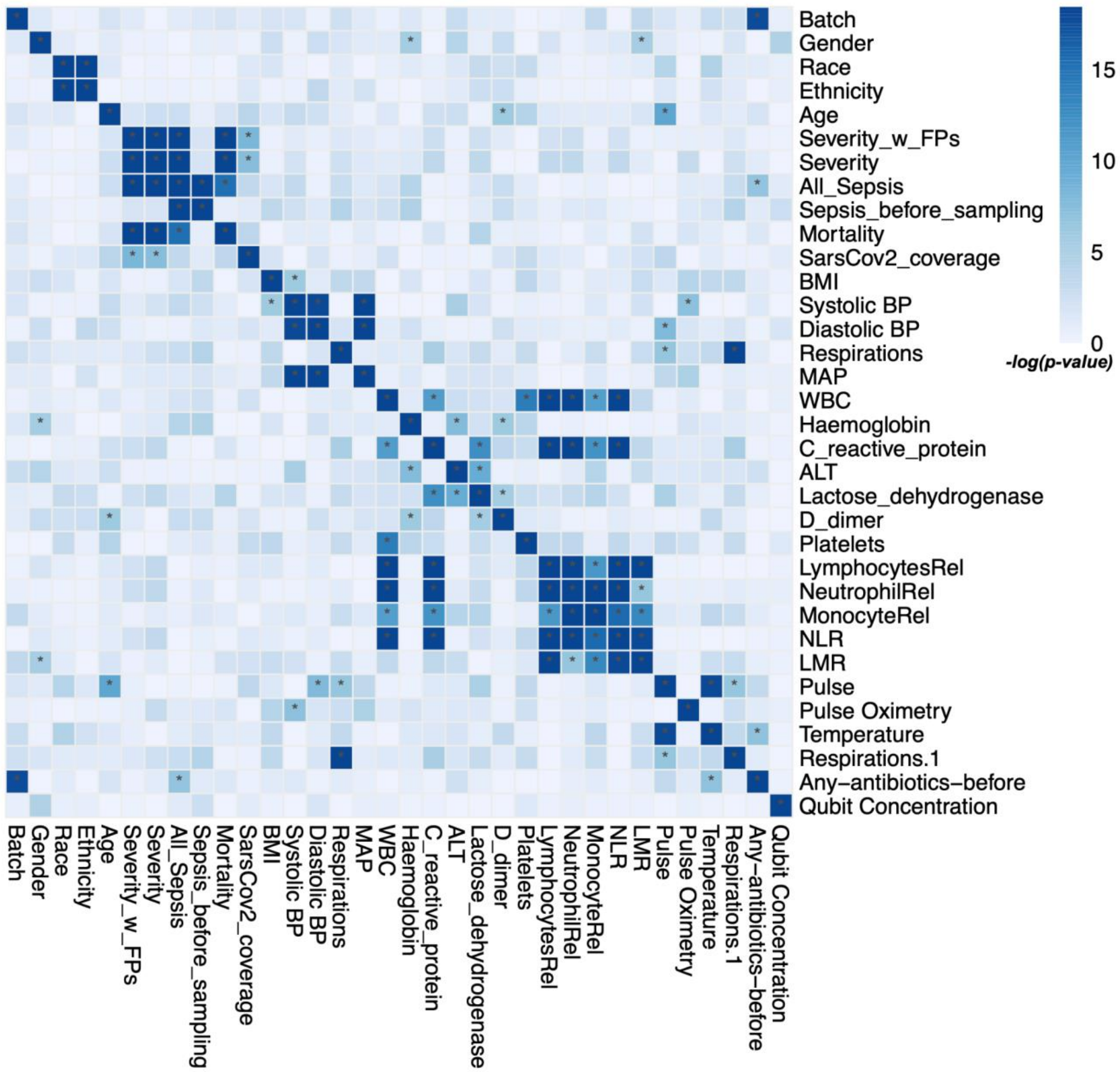

Figure S2. Collinearity between metadata variables including clinical and patient characteristics. * refers to p-value<0.005. -log(p-value) of feature correlation has been calculated with three different tests based on data types (Kruskal-Wallis, Spearman, Chi-Squared). For model verification, we observe that race and ethnicity, as well as severity and mortality, as expected, are significantly correlated.

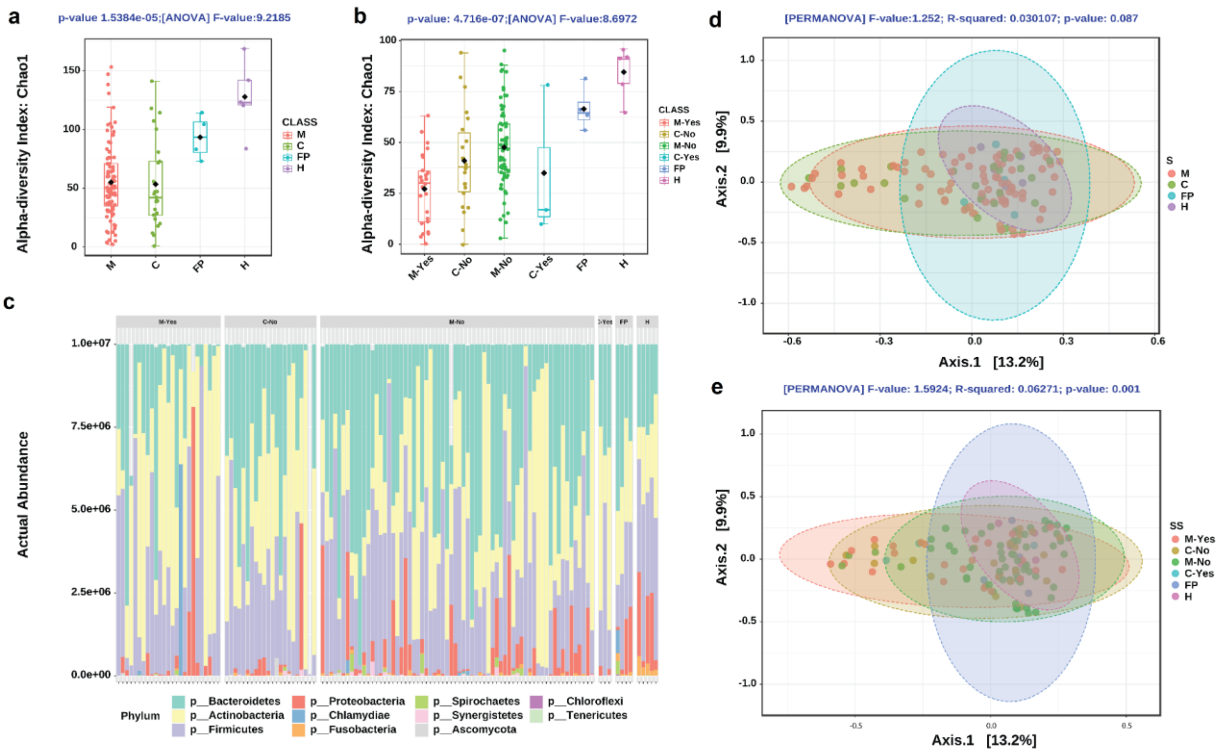

Figure S3. a. Alpha diversity boxplots for four groups: moderate (M) and critical (C) COVID-19 groups, false positives (FP), and healthy/control (H) groups. **b.** Alpha diversity boxplots with COVID-19 groups stratified by BSA timing [BSA before or same day as saliva sampling: Yes (Y), No (N)]. **c.** Phyla Abundance Plot. **d.** Beta diversity PCA graphs for the four groups. **e.** Beta diversity PCA graphs with COVID-19 groups stratified by BSA timing.

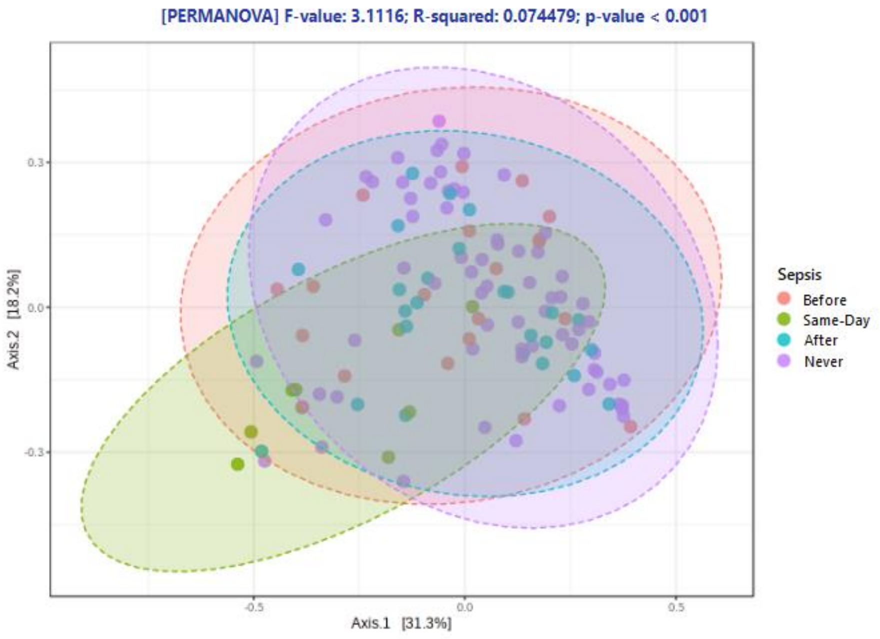

Figure S4. Bacterial Microbiome Beta Diversity at the Genus Level

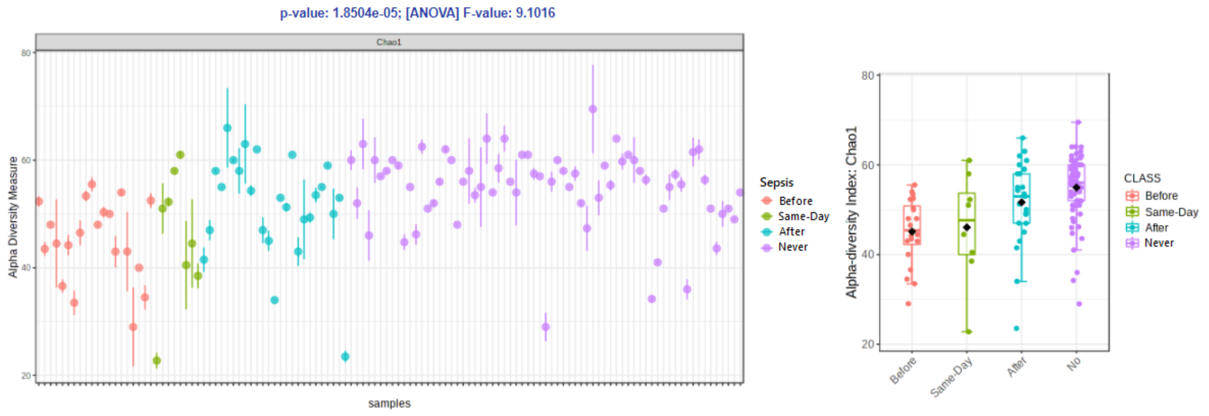

Figure S5. Bacterial Microbiome Alpha Diversity at the Genus Level

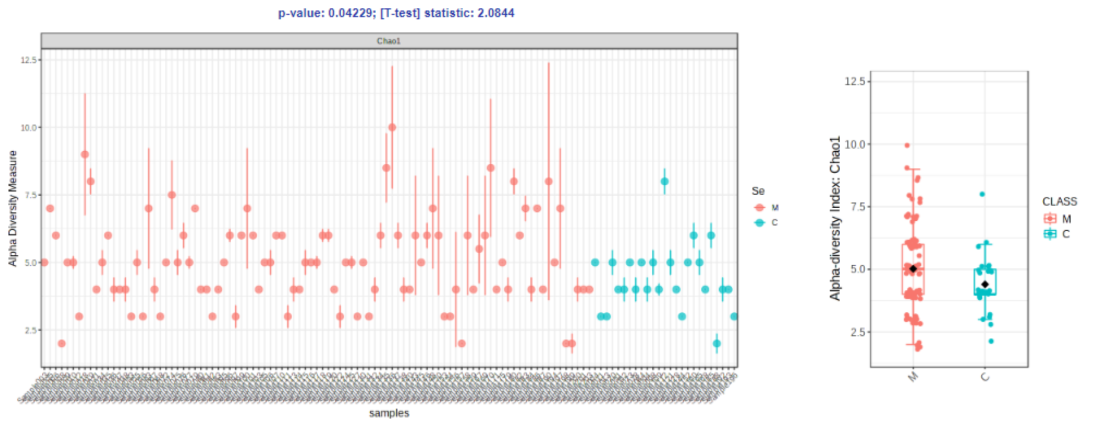

Figure S6. Alpha Diversity of DNA Virus, Fungi and Parasite at the family level by Disease Severity groups (M: Moderate, C: Critical)

a

b

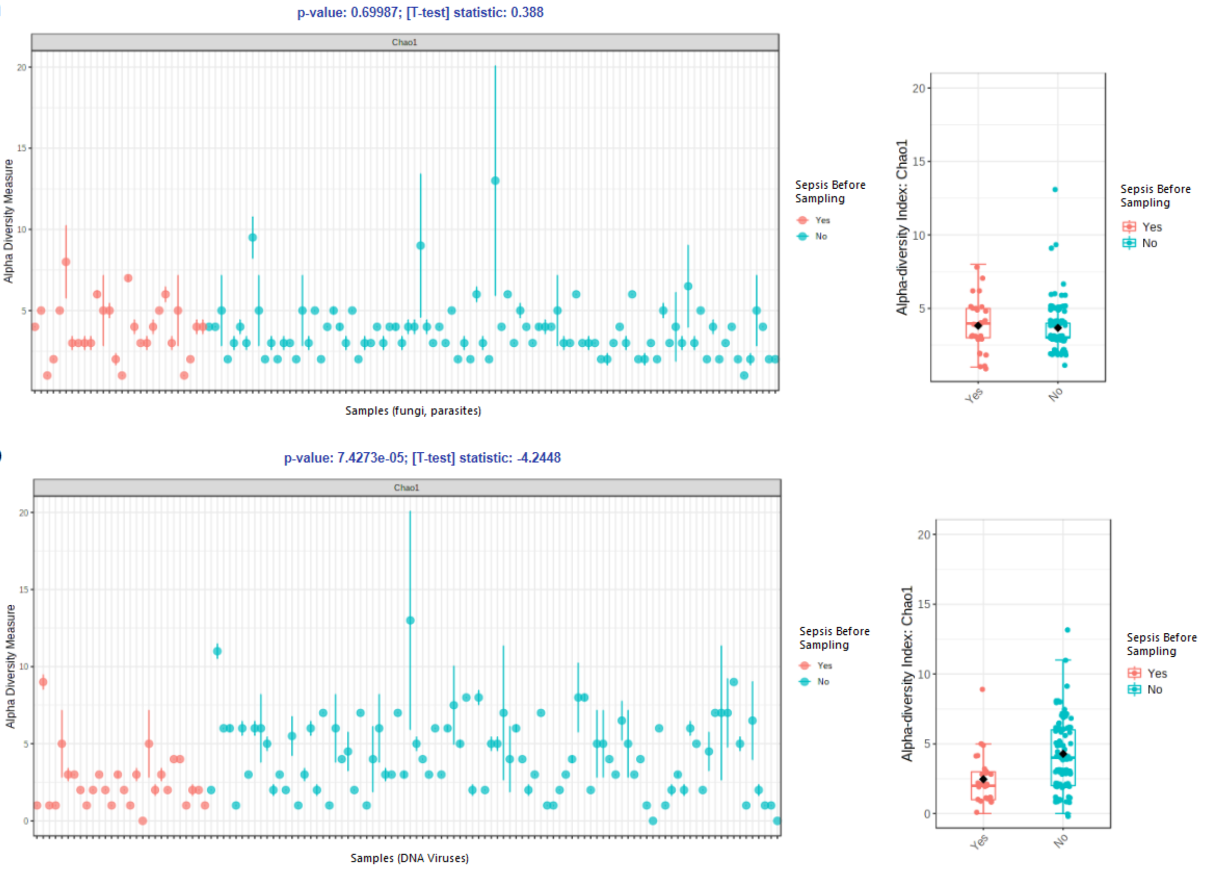

Figure S7. a Alpha Diversity of Fungi and Parasite Species by BSA groups (Yes: Early BSA, No: Early BSA) **b** Alpha Diversity of DNA Virus Species by BSA groups

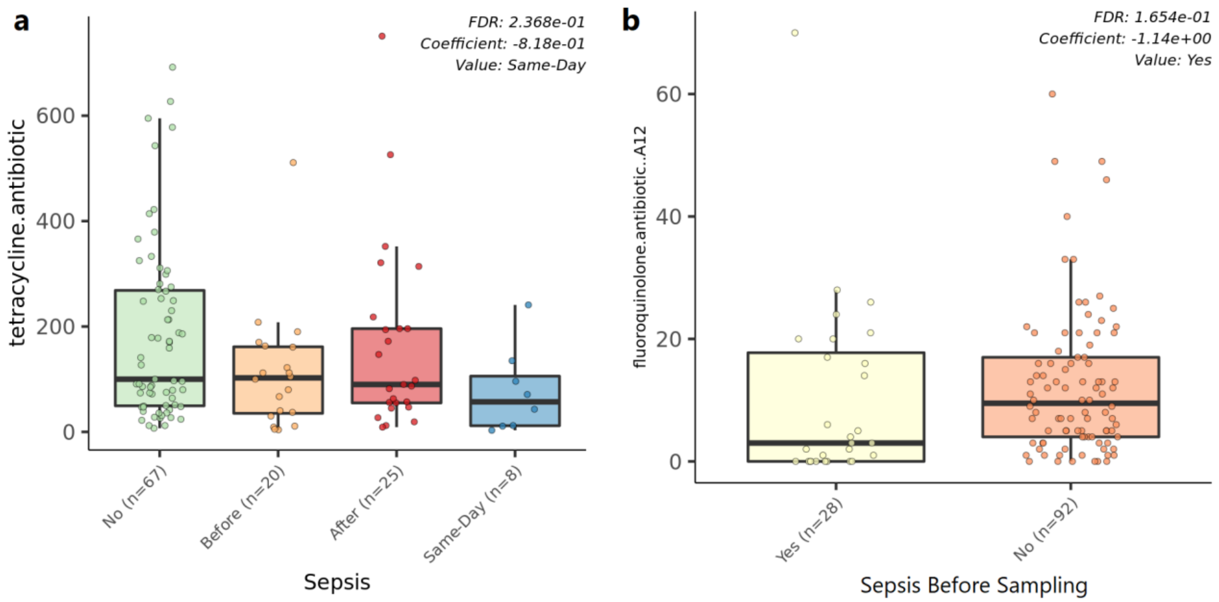

Figure S8. a Maaslin Boxplots showing abundance of genes with tetracycline resistance among the 4 sepsis BSA groups (Early BSA: Before and Same-Day), **b** Maaslin Boxplots comparing mixed class fluoroquinolone and tetracycline (A12) resistance to among the 2 sepsis BSA groups (Yes: Early BSA, No: No Early BSA)
